# Cervical Ripening Balloon in Combination with Methotrexate and Potassium Chloride for the Treatment of a 13-week Cervical Ectopic Pregnancy

**DOI:** 10.1155/2023/4725663

**Published:** 2023-05-02

**Authors:** Alina Tvina, Emily Smith, Meredith Cruz

**Affiliations:** ^1^Department of Obstetrics and Gynecology, Medical College of Wisconsin, Milwaukee, WI, USA; ^2^Division of Maternal Fetal Medicine, Medical College of Wisconsin, Milwaukee, WI, USA

## Abstract

**Background:**

Cervical pregnancy, an uncommon type of ectopic pregnancy, can lead to devastating consequences if not diagnosed and treated early. Despite this, there are no specific guidelines on how to treat such pregnancies especially in advanced gestational ages (GAs).

**Case:**

This is a 35-year-old patient who presented to our hospital at 13 weeks GA after failing systemic multidose methotrexate therapy for a cervical ectopic pregnancy. Given desire to preserve fertility, a minimally invasive conservative approach was taken involving potassium chloride (KCl) and methotrexate injections into the gestational sac, followed by immediate Cook intracervical double balloon placement under direct ultrasound visualization, with removal of the balloon after 72 hours, and ultimately resolution of the pregnancy 12 weeks after the removal.

**Conclusion:**

Advanced first trimester cervical ectopic pregnancy after failure of methotrexate therapy was managed successfully with minimally invasive KCl and methotrexate injections in combination with cervical ripening balloon.

## 1. Introduction

Cervical pregnancy is the rarest kind of ectopic pregnancy representing <1% of all ectopic pregnancies [1]. It has an unknown etiology and potentially life-threatening consequences if not diagnosed and treated early. Most cases reported suggest variety of minimally invasive treatments, yet there is no clear guidance on the preferred method of treatment, especially with advanced gestational age (GA). We present a patient with a late first trimester cervical pregnancy at 13-week GA status post-treatment failure with systemic methotrexate who desired fertility preservation.

## 2. Case

**Figure 1:**
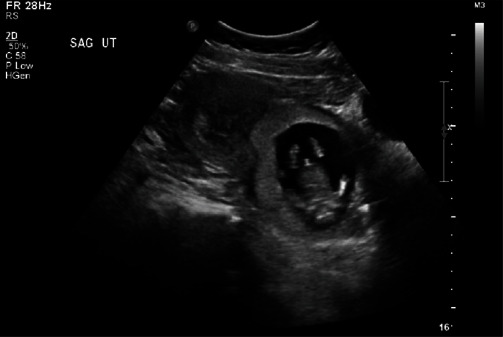
Transabdominal and sagittal ultrasound view demonstrating cervical pregnancy with empty endometrial cavity.

A 35-year-old G2P0010 woman with a history of a first trimester spontaneous miscarriage presented to the emergency department at 7 weeks and 3 days GA dated by her last menstrual period with vaginal bleeding. Transvaginal ultrasound demonstrated a gestational sac (GS) with an embryo and crown rump length correlating with 7 weeks 6 days GA implanted in the cervical stroma (Figure 1). Serum beta human chorionic gonadotropin levels (*β*-HCG) were 89,312 mIU/ml at presentation. The patient received a 50 mg/m^2^ dose of methotrexate Intramuscular (IM), with a second dose administered on day 7 from first dose given increasing levels of serum *β*-hCG. Third and fourth doses were administered on days 12 and 15 from first dose for insufficient decrease in *β*-HCG levels of less than 15% between days 7 and 11, and subsequent up trending of the hormone level on day 15.

**Figure 2:**
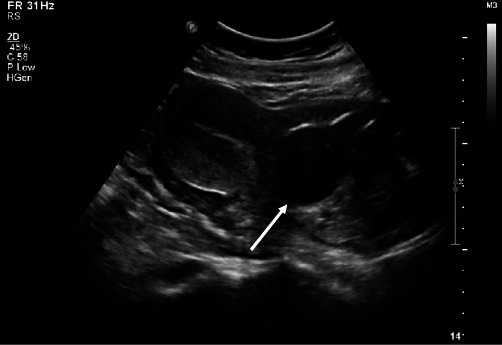
Transabdominal and sagittal ultrasound view demonstrating the Cook intracervical ripening balloon compressing the GS (arrow).

**Figure 3:**
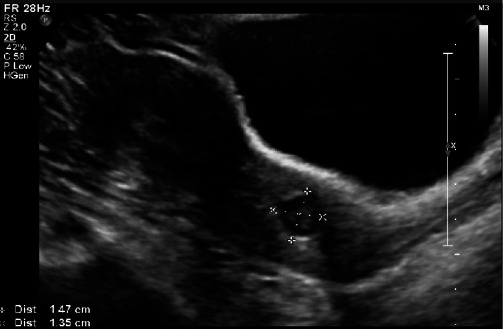
Transabdominal and sagittal ultrasound 12 weeks after removal of balloon demonstrating GS decreased to 1.4 cm.

The patient was then rereferred to our health center at 13-week GA for further management. A *β*-hCG level at that time measured was 84,984 mIU/ml. Given advanced GA and patient's desire to preserve future fertility, the decision was made to proceed with medical treatment in combination with cervical ripening balloon under general anesthesia. A 5″, 17-G epidural needle was inserted into the GS under transabdominal ultrasound guidance and advanced to the fetal heart. Five milliequivalent (2.5 ml) of potassium chloride (KCl) was injected intracardially. After cessation of fetal cardiac activity, 1 mg/kg (80 mg) of methotrexate was injected into the GS. GS diameter was measured 8.5 cm at the time of the procedure. Immediately after the injections, a Cook cervical ripening double-balloon catheter was placed through the cervix and into the uterus under ultrasound guidance. The uterine balloon was filled with 30 ml of normal saline. The vaginal balloon was inflated to 60 ml in the cervix directly over the GS while visualizing compression of the GS on transabdominal ultrasound (Figure 2). 72 hours after the procedure, the cervical ripening balloon was removed under ultrasound guidance. The GS noted to have decreased in size to 6.7 cm with *β*-hCG level declined to 29,160 mIU/ml. Patient was discharged home, and was monitored weekly with ultrasounds demonstrating gradual decrease in GS diameter. Three weeks after ripening balloon removal, the GS measured to be 6.5 cm and the patient reported to have passed pregnancy tissue at home. GS diameter measured 4 cm at 7 weeks, and finally, 1.4 cm at 12 weeks (Figure 3) with nonpregnant *β*-hCG levels (<5 mIU/ml). Patient was instructed to follow-up as needed.

## 3. Discussion

Cervical pregnancy is a rare, and high-risk ectopic pregnancy with incidence of 1 : 1,000–95,000 pregnancies [2]. It is diagnosed using a transvaginal ultrasound demonstrating the placenta and entire chorionic sac containing the pregnancy below the internal cervical os and can be distinguished from a spontaneous abortion in process by the presence of embryonic cardiac activity or presence of a rich vascular pattern around the GS [3]. Presenting symptoms generally include vaginal bleeding and can be coupled with abdominal pain, particularly in more advanced GAs [4]. The etiology of a cervical pregnancy is unknown; however, risk factors associated with increased incidence include prior uterine curettage, Asherman's syndrome, induced abortion, in vitro fertilization, presence of leiomyomas, presence of intrauterine device, and prior cesarean section [5, 6]. Early diagnosis and management are crucial, given cervical pregnancy is life threatening and can lead to a massive hemorrhage that may require hysterectomy and blood transfusion for life saving purposes. Given the rarity of this condition, there is no consensus on the preferred way of treatment for different GAs. Traditionally, cervical pregnancies were treated with hysterectomy. However, with earlier diagnosis more and more minimally invasive and conservative treatments are being offered to patients who desire to preserve future fertility.

Vela and Tulandi reported 12 cases of cervical ectopic pregnancies, where pregnancies of 12–16 weeks gestation age were treated with curettage and cervical suturing requiring subsequent hysterectomy for uncontrolled bleeding [7]. Fylstra reported 13 cases treated with curettage and an immediate post curettage cervical canal balloon placement for cervical pregnancies up to 12 weeks gestation [8]. Jeng et al. reported a case series of 38 cervical pregnancies with mean GA of 8 6/7 weeks terminated with ultrasound guided methotrexate injection into the GS and additional intracardiac fetal KCl injection to pregnancies with evident cardiac activity [9]. Similar to Jeng et al., Timor-Tritsch et al. reported termination of five cervical pregnancies 6–8 weeks gestation with ultrasound guided injection of methotrexate into the GS [3]. Timor-Tritsch et al. also reported successfully treating seven cesarean scar and three cervical ectopic pregnancies with GAs ranging from 6 3/7 to 7 4/7 weeks GA via cervical ripening balloon placement [10]. García et al. described four cases of cervical ectopic pregnancies treated conservatively with a combination of methotrexate and mifepristone. Three of the four cases ultimately required subsequent intervention with either uterine artery embolization, dilation, and curettage or vaginal misoprostol. Of note, unlike our case, all the above cases were treated at an early GA prior to 9 weeks [11]. Tanos et al. reported four cases of cervical ectopic pregnancy treated solely by operative hysteroscopy. All cases were less than 8 weeks GA [12].

The case, we described above, presents a clinical challenge given management of cervical ectopic pregnancies with advanced gestation has not been well described in the literature. In our attempts to provide adequate care for the patient with the goal of avoiding major surgery, we describe a unique therapy combination of ultrasound guided KCl fetal intracardiac injection in combination with intragestational sac methotrexate injection, followed by immediate intracervical double balloon catheter placement for 72 hours. This method was successful at treating this late first trimester cervical pregnancy with resolution of the pregnancy measured by serial ultrasounds and serum *β*-hCG levels obtained 12 weeks after the removal of the cervical balloon. We believe that this combination technique, being first described here with advanced GA, can be offered to hemodynamically stable patients with cervical pregnancies who failed systemic methotrexate therapy and desire to preserve future fertility.

## Data Availability

Data sharing not applicable to this article as no datasets were generated or analyzed during the current study.
